# Sensitive pH Monitoring Using a Polyaniline-Functionalized Fiber Optic—Surface Plasmon Resonance Detector

**DOI:** 10.3390/s21124218

**Published:** 2021-06-19

**Authors:** Iulia Antohe, Luiza-Izabela Jinga, Vlad-Andrei Antohe, Gabriel Socol

**Affiliations:** 1 National Institute for Laser, Plasma and Radiation Physics (INFLPR), Atomiştilor Street 409, 077125 Măgurele, Ilfov, Romania; iulia.antohe@inflpr.ro (I.A.); izabela.jinga@inflpr.ro (L.-I.J.); 2 Faculty of Physics, R&D Center for Materials and Electronic & Optoelectronic Devices (MDEO), University of Bucharest, Atomiştilor Street 405, 077125 Măgurele, Ilfov, Romania; vlad.antohe@fizica.unibuc.ro; 3 Institute of Condensed Matter and Nanosciences (IMCN), Université Catholique de Louvain (UCLouvain), Place Croix du Sud 1, B-1348 Louvain-la-Neuve, Belgium

**Keywords:** fiber optic–surface plasmon resonance (FO-SPR) sensors, Platinum (Pt) thin film, Polyaniline (PANI) electroless synthesis, pH monitoring, pH sensitive polymer

## Abstract

In this work, we report results on the fabrication and characterization of a surface plasmon resonance (SPR) pH sensor using platinum (Pt) and polyaniline (PANI) layers successively coated over an unclad core of an optical fiber (FO). The plasmonic thin Pt layer was deposited using a magnetron sputtering technique, while the pH-sensitive PANI layer was synthesized using an electroless polymerization method. Moreover, the formation of PANI film was confirmed by X-ray photoelectron spectroscopy (XPS) technique and its surface morphology was investigated using scanning electron microscopy (SEM). It was found that the PANI/Pt-coated FO-SPR pH sensor exhibits a fast and linear response in either acid or alkali solutions (pH operational range: 1 to 14). The proposed FO-SPR sensor could be used for biomedical applications, environmental monitoring or any remote, real-time on-site measurements.

## 1. Introduction

The measurement and control of pH are crucial in the fields of biochemistry, clinical medicine, agriculture, food processing, and environmental science, to name a few [1,2]. Consequently, various pH sensing techniques based on optical, electrochemical, or physicochemical methods have been developed. In the last decade, advanced sensing materials including metal, metal oxides, polymers, semiconductors, as well as nanoparticles and nanotubes, were used to build cost-effective and user-friendly integrated pH monitoring systems [3]. However, the conventional and widely employed method for pH measurement uses a pH glass electrode and potentiometric technique. Unfortunately, these sensors are bulky in size, they often exhibit a rather poor performance in both extremes of the pH scale and they also have to be re-calibrated in a discontinuous way [4]. In recent years, substantial efforts were invested towards the development of optical fiber-based pH sensors [4,5,6].

In this context, fiber optic–surface plasmon resonance (FO-SPR) pH sensors have attracted increasing interests due to their many advantages, such as low-cost production, small size, immunity to electromagnetic interferences, remote sensing capability, and the safety for *in-vivo* measurement [7,8,9,10]. In the FO-SPR technology, the light is guided through a metal (typically gold)-coated multimode optical fiber to yield propagating plasmonic waves at the interface obtained with the analyzing environment (typically a liquid). Sensitive changes in the refractive index (RI) of light are then triggered by any biological interaction or chemical event occurring at this interface, and they can be further processed into a real-time monitored graphical representation [11,12,13,14]. In 2012, S. Singh et al. proposed a transmission type FO-SPR pH sensor prepared using three consecutive layers of silver, silicon, and a pH-sensitive hydrogel [15]. The change in pH around the sensor induced hydrogel swelling or shrinkage resulting in the change of its RI. This sensor operated in the low and high pH range (from 3 to 12) and had a fast response time (around 1 min). One year later, S.K. Mishra reported a Ag/ITO/Al/hydrogel layers-based FO-SPR pH sensor operating in good conditions only in a particular window, for higher and lower pH values, with detection capability issues for the range of middle pH values [16]. In 2017, A.L. Aldaba et al. fabricated polyaniline (PANI)-coated tilted fiber Bragg gratings for pH sensing varying from 2 to 12 [7]. PANI presence rendered these sensors to exhibit a faster response when compared to a common digital pH-meter and a good degree of repeatability. More recently, PANI was used in combination with graphene oxide to fabricate a highly-sensitive transmission-based FO-SPR pH system, operating in a pH sensing range from 2.4 to 11.35 [17]. However, there is no evidence of utilizing reflection-type FO-SPR sensors for pH detection, neither based on hydrogel or PANI polymers. Reflection-type FO-SPR sensors are in general more suitable for *in-situ* sensing applications and more reliable because the exposed optical fiber tip is guaranteed to be strain and bend-free, being, thus, much less susceptible to stress-induced breakage [13]. Nevertheless, despite the obvious advantages of this technology, the applicative range of these devices is typically limited by the conventional Au plasmonic film predominantly used so far [11,14].

In this work, we report results on the fabrication and characterization of a reflection-type FO-SPR pH sensor built by coating platinum (Pt) and electro-conductive PANI layers over the unclad core of an optical fiber tip. The thin Pt layer was deposited using a magnetron sputtering technique, while the pH-sensitive PANI layer was synthesized using an electroless polymerization method. Pt was thus used as a plasmonic material within such a reflection-type FO-SPR sensor due to its superior catalytic properties, essential for subsequent PANI synthesis steps [18]. PANI is a well-known electro-conductive polymer with excellent stability and physico-chemical properties, featuring rapid and reversible adsorption and desorption *RedOx* kinetics [19,20]. In these circumstances, PANI can be successfully employed as a functional sensitive layer in energy storage applications [21], pollutants detection [19,22], gas sensing [23], and pH monitoring [24,25]. To the best of our knowledge, few theoretical studies were reported with Pt-coated transmission-type FO-SPR sensors [26,27]. Only in our recent work, a Pt-coated reflection-type FO-SPR sensor has been proposed for the first time, wherein the thin Pt plasmonic film has been used in conjunction with a functional PANI film to demonstrate highly-sensitive detection capabilities of the 4-nitrophenol pollutant [22]. Herein, we continued the optimization studies of the novel Pt/PANI bilayer reflection-type FO-SPR sensor, aiming to maximize its bulk sensitivity and to expand its applicative range towards pH monitoring. In particular, the formation of PANI film on the Pt-coated FO-SPR surface was complementarily evaluated by X-ray photoelectron spectroscopy (XPS), while its surface morphology was investigated by scanning electron microscopy (SEM). The PANI/Pt-coated FO-SPR pH sensor was afterwards characterized and noteworthy, it exhibited an increased bulk sensitivity compared to our previous report, as well as a fast and linear response in either acid or alkali solutions (pH operational range: 1 to 14). This optical FO-SPR sensor configuration might have a great potential in pH monitoring-based applications, since it is linearly responsive to a wide pH range compared with other pH measuring technologies, it has high sensitivity, it can be fabricated in simple steps, it has reduced dimensions and it offers the possibility for out-of-the-lab on-site measurements.

## 2. Materials and Methods

### 2.1. Reagents and Materials

Deionized water (DIW) purified by a Milli-Q 50 system (TKA, Germany) was employed in all experiments. Acetone, sodium hydroxide (NaOH), potassium phosphate dibasic (K${}_{2}$HPO${}_{4}$), sodium chloride (NaCl), sulfuric acid (97% H${}_{2}$SO${}_{4}$), D(+)-sucrose, ethanol, and aniline (99% C${}_{6}$H${}_{5}$NH${}_{2}$) were bought from Merck. The nitrogen and oxygen gas 6.0 purity bottles were provided by Messer. The multimode optical fiber of 400 μm diameter was purchased from Thorlabs. The Pasteur glass pipettes, the double wall glass, and the magnetic stir plate were supplied by VWR.

### 2.2. FO-SPR Sensing Device

**Figure 1:**
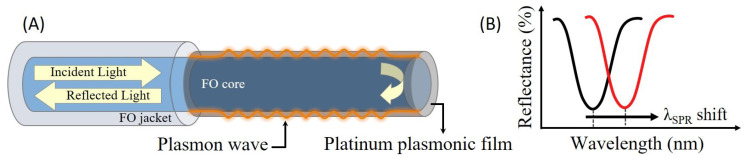
Reflection-type FO-SPR sensor’s working principle. (**A**) Schematic of the Pt-coated FO-SPR sensor, where the detection occurs by monitoring the light reflected back by the optical fiber mirror-like tip (backward scattering mode). (**B**) Simulated SPR spectral dip response, where the wavelength is right-shifting as a function of local RI changes at the sensor surface.

The FO-SPR sensing platform and its working principle (see Figure 1) were presented in more details elsewhere [14,22]. Briefly, herein, a sensitive SPR zone of 0.6 cm was created at one side of a multimode optical fiber (with a total length of 3.6 cm and a diameter of 400 μm) by removing the jacket and subsequently uncladding the optical fiber in acetone (Figure 1A). Afterwards, the exposed silica core of the optical fiber was isotropically coated by a thin Pt film (40 nm), using a sputter coater (Quorum Q150R ES, UK). The DC plasma was applied for 15 min at 54 mA in Ar atmosphere kept at $2.5\times 10^{-2}$ mbar. During the sputtering process, the FO-SPR sensors were installed on a rotating stage (100 rpm) to improve the Pt coverage on the optical fibers, while the deposited thickness was estimated using the built-in quartz crystal microbalance (QCM) oscillator system. In this reflection-type FO-SPR configuration, RI changes occurring at the plasmonic interface can be observed by monitoring the SPR wavelength shifts within the reflectance spectra, as schematically represented in Figure 1B.

### 2.3. FO-SPR RI Measurements

The bulk sensitivity of the Pt-coated FO-SPR sensors was determined by refractive index (RI) measurements in a serial set of sucrose dilutions (0, 2, 4, 8, 12% *w*/*w*). The Brix values of these sucrose dilutions were verified with a digital refractometer (Atago Palette PR-32, Japan) and the corresponding RI units were reported elsewhere [14,22]. The interchangeable FO-SPR sensor was inserted into the connector and fixed on the computer-controlled SPR sensing platform (see Section 2.2). The metal-coated FO-SPR sensor was then held one minute in each sucrose solution during the RI measurements. The FO-SPR bulk sensitivity was next determined by plotting the SPR wavelength shifts as a function of the RI values of each sucrose solution, pursued by linearly fitting the obtained calibration curve. Moreover, the figure of merit (FOM) was also evaluated by making the ratio between the wavelength shift sensitivity (S) and the linewidth of the spectral resonance dip, given as the full width at half-maximum (FWHM). The FWHM value was extracted by using the data analysis and graphing Origin (OriginLab) software.

### 2.4. pH Monitoring Using the PANI/Pt-Coated FO-SPR Sensors

The prepared Pt-coated FO-SPR sensors were further used as catalysts in the electroless polymerization of aniline. The rudimentary steps of PANI electroless deposition approach were selected from literature [18,28] and optimized to the micrometer-sized optical fiber geometry [22]. Briefly, the Pt-coated substrates were immersed in an aqueous solution of aniline (0.4 M) and H${}_{2}$SO${}_{4}$ (0.4 M) for 6 h. This solution was kept under oxygen saturation and at a constant temperature of $25{\;}^{\circ }$C by means of a thermostatic circulating water bath model TC120 from Grant Instruments, for reproducibility reasons. Subsequently, a greenish film gradually appeared on the Pt surface. After 6 h, the samples were taken out of the reactor, carefully rinsed with DIW and then the PANI film was undoped by immersing the sensors in a 1 M NH${}_{4}$OH solution for 30 min. The as-prepared PANI/Pt-coated FO-SPR sensors were next used for direct detection of different pH values ranging from 1 to 14 (aqueous solutions) while keeping a constant temperature of $25{\;}^{\circ }$C for preventing the temperature-induced pH fluctuations of the solutions to affect the measurements. Each pH was measured three times using PANI/Pt FO-SPR sensors. The pH solutions ranging from 1 to 14 were prepared in a NaCl buffer by adding NaOH or HCl. Accurate pH values of each solution were determined with a Consort C1010 pH-meter with a resolution of 0.01 pH. As reported elsewhere, the reagents used for preparing the pH analyzing media have been specifically selected to minimize the potential disturbances caused by the RI variation of the pH solutions [7,29]. Their RI units were thus verified with the digital refractometer, and the measured value of ∼1.334 (closed to the typical RI value of DIW, i.e., 1.333) was considered constant among the pH solutions, as it lied within the resolution of the digital refractometer.

### 2.5. Observations of the FO-SPR Surfaces

A field emission–scanning electron microscope (FE-SEM, JEOL7600F) was used to examine the surface morphology of the PANI film synthesized on the Pt-coated FO-SPR sensors. Notably, the SEM analysis was performed onto Pt-coated FO-SPR sensors covered by PANI films deliberately doped (protonated) in a 1 M HCl solution for 10 min to enhance their conductivity and, thus, to further reduce specimen charging effects during the SEM observations. X-ray photoelectron spectroscopy (XPS) measurements were performed on a Thermo Fisher Scientific ESCALAB Xi+ setup equipped with a multichannel hemispherical electron analyzer (dual X-ray source) working with Al${}_{K_{\alpha }}$ radiation (1486.2 eV). The binding energies were determined by reference to the C-(C,H) component of C 1s peak set at 284.8 eV. XPS data were recorded on the PANI/Pt-coated FO-SPR sensors previously outgassed in the pre-chamber of the setup at room temperature at a pressure <$2\times 10^{-6}$ Pa to remove the chemisorbed water from their surfaces. The surface chemical composition and oxidation states of PANI were estimated from the XPS spectra by calculating the integral of each peak after subtraction of the “S-shaped” Shirley-type background using the appropriate experimental sensitivity factors by means of Avantage Software version 5.978. The XPS measurements were carried out on basified (undoped) PANI, whilst the XPS spectra were analyzed using NIST X-ray Photoelectron Spectroscopy Database and The Handbook of X-ray Photoelectron Spectroscopy by J.F. Moulder et al. [30].

## 3. Results and Discussion

### 3.1. Platinum-Coated FO-SPR Sensor Bulk Sensitivity

**Figure 2:**
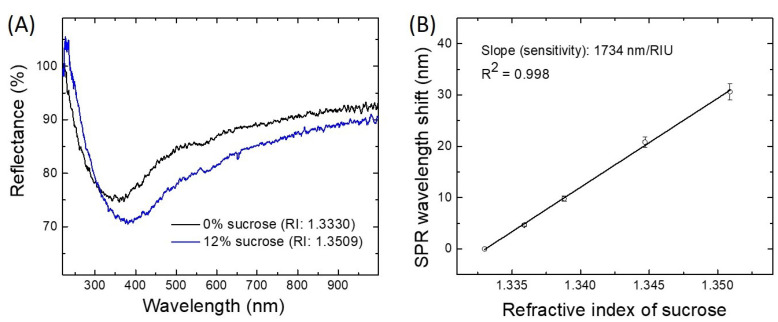
Evaluation of the Pt-coated FO-SPR sensors’ performance. (**A**) Typical SPR spectral dips obtained in 0% (black) and 12% (blue) sucrose dilutions using Pt-coated FO-SPR sensors; (**B**) The corresponding calibration curve measured in sucrose dilutions (0, 2, 4, 8 and 12% *w*/*w*) with the Pt-coated FO-SPR sensors. The error bars represent standard deviation (n=3). $R^{2}$ denotes the coefficient of determination.

Three Pt-coated FO-SPR sensors were prepared and their performance indicators (i.e., bulk sensitivity—S, and figure of merit—FOM) were determined. The bulk sensitivity (S) is expressed as the ratio between the wavelength shift ($\Delta \lambda_{\mathrm{SPR}}$) and the RI change (Δn) of the analyzing medium: $S=\Delta \lambda_{\mathrm{SPR}}/\Delta n$. The bulk sensitivity of the Pt-coated reflection-type FO-SPR sensors was evaluated by performing RI measurements in sucrose dilutions (0, 2, 4, 8, 12% *w*/*w*). Figure 2A illustrates the SPR spectral dips obtained in 0% (DIW) and 12% sucrose dilutions using Pt-coated sensors. The obtained SPR shifts were plotted as a function of RI for generating the calibration curve displayed in Figure 2B. As can be observed, the Pt-coated FO-SPR sensors have a sensitivity of 1734 nm/RIU. Moreover, the FOM=S/FWHM [${\mathrm{RIU}}^{-1}$] of the Pt-coated FO-SPR sensors was determined to be around 9 ${\mathrm{RIU}}^{-1}$.

Noteworthy, in terms of bulk sensitivity, the Pt-coated reflection-type FO-SPR sensors compete with the classical Au-coated FO-SPR devices [12,22]. However, the FOM was lower mainly due to a higher FWHM value of the SPR spectral dips. Nevertheless, despite the latter observation, the obtained results are promising as the Pt-based FO-SPR sensors may expand the applicative range of these optical devices, by benefiting from the excellent catalytic activity and stability of Pt, or by allowing measurements in a lower wavelength spectral window.

### 3.2. Morpho-Structural Characterization of PANI/Pt-Coated FO-SPR Sensor’ Surfaces

**Figure 3:**
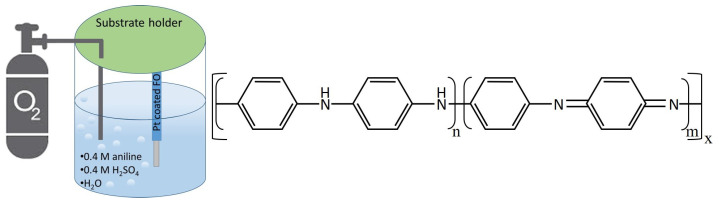
Schematic of PANI synthesis protocol and its different oxidation states. In the case of Pernigraniline (oxidized state) n=0 and m=1; Leucoemeraldine (reduced state) n=1 and m=0; Emeraldine (mid *RedOx* state) n=m=0.5.

PANI thin films were synthesized on the Pt-coated FO-SPR substrates using an electroless deposition method well described in literature [18,28]. This polymerization approach was adapted to the FO-SPR sensor’ architecture and the process parameters (oxygen flow, bath temperature, process duration) have been previously optimized elsewhere to obtain an adequate PANI thickness around 95 nm [22], allowing an excellent SPR response in aqueous solutions. In brief, PANI is simply obtained through the polymerization of aniline on the Pt surface acting as a catalyst. The process is based on spontaneous chemical reactions in acidic medium, involving reduction in dissolved oxygen as cathodic half-reaction and oxidation of aniline as anodic half-reaction at the metal–solution interface [18]. The polymerization reaction is thus initiated on the Pt surface by a catalytic oxygen reduction, and then the primary formed PANI layer takes over the autocatalytic polymerization of aniline. Consequently, when the Pt-coated FO-SPR sensors were immersed in the electroless reactor kept under oxygen saturation, a light greenish colour gradually appeared on their surface. The greenish colour appearance is a characteristic of the acidified (doped) Emeraldine mid *RedOx* state of PANI. PANI has different oxidation states as shown in Figure 3. The stable-in-air basified (undoped) form of PANI, known as Emeraldine (light green), can be reduced to Leucoemeraldine (white-yellow) or oxidized to Pernigraniline (dark blue).

PANI is an excellent electro-conductive polymer to be used in pH sensing applications, and particularly when coupled to the FO-SPR sensing technology, because it changes its chemical structure in both, acidic and alkaline medium, causing further modifications of its electrical and optical properties, including its refractive index [8].

**Figure 4:**
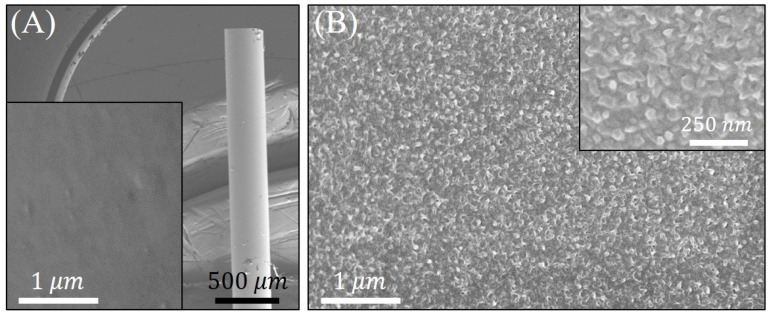
(**A**) Low-magnification SEM micrograph of a FO-SPR fiber tip, with the inset showing its Pt-coated surface before the PANI deposition process; (**B**) Corresponding FO-SPR sensor surface after PANI electroless synthesis for 6 h at $25{\;}^{\circ }$C. The inset of (**B**) represents a magnified SEM image of the PANI/Pt-coated FO-SPR sensor surface.

SEM micrographs attained from the surface of a Pt-coated FO-SPR sensor before and after PANI electroless deposition for 6 h, at $25{\;}^{\circ }$C are presented in Figure 4. As noticed, the Pt coating on the FO-SPR surface is homogenous and smooth (inset of Figure 4A). Furthermore, the PANI film on the Pt-coated FO-SPR sensor (Figure 4B) can be identified by its rough aspect (see also the inset of Figure 4B), resembling a typical morphology of a thin PANI film deposited on a Pt substrate through an electroless method [18,31]. The synthesized PANI film was consistent and well adhered to the Pt-coated FO-SPR sensor surface.

**Figure 5:**
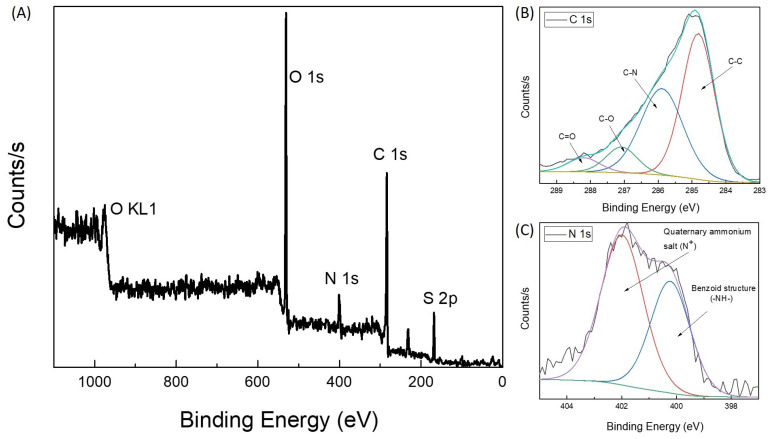
XPS spectra: (**A**) Wide scan of electroless-deposited PANI film; PANI C 1s (**B**) and N 1s (**C**) core-level spectra of the film obtained by electroless synthesis.

Corresponding XPS spectra of the FO-SPR surface presented in Figure 4B are shown in Figure 5. The wide scan of an electroless deposited PANI film obtained after 6 h of reaction at $25{\;}^{\circ }$C is presented in Figure 5A. The wide scan shows three peaks attributed to C 1s (284.8 eV), N 1s (398 eV), O 1s (532 eV) an S 2p (170 eV). The carbon (C) and nitrogen (N) signals are due to the PANI presence on the Pt-coated FO-SPR surface. The oxygen (O) signal is attributed to PANI after oxidation. Figure 5B,C displays, respectively, the C 1s and N 1s core-levels spectra of the film. The N 1s spectrum can also be deconvoluted into the usual PANI component peaks at 398.2 eV and 399.4 eV, attributed to the imine (-N=) and amine (-NH-) groups, respectively, with a higher binding energy (>401 eV) tail due to oxidized species or positively charged N. The presence of sulfur (S) was also determined in the analyzed sample due to the traces of sulfate (168.96 eV) and sulfuric acid (170.21 eV) used in the electroless polymerization process of aniline.

### 3.3. pH Measurements Using PANI/Pt-Coated FO-SPR Sensors

**Figure 6:**
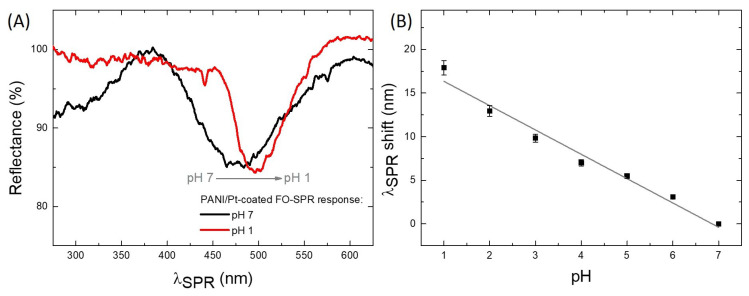
Graphical representation of the SPR wavelength shift as a function of acidic pH values. (**A**) The SPR spectral dips obtained in solutions of pH 7 (black) and pH 1 (red), respectively; (**B**) FO-SPR sensor linear calibration curve for pH values ranging from 1 to 7. The error bars represent standard deviation (n=3). The regression line has a coefficient of determination ($R^{2}$) of 0.95.

**Figure 7:**
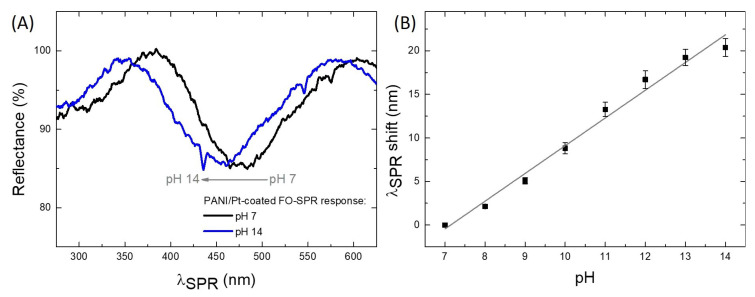
Graphical representation of the SPR wavelength shift as a function of alkaline pH values. (**A**) The SPR spectral dips obtained in solutions of pH 7 (black) and pH 14 (blue), respectively; (**B**) FO-SPR sensor linear calibration curve for pH values ranging from 7 to 14. The error bars represent standard deviation (n=3). The regression line has a coefficient of determination ($R^{2}$) of 0.98.

The freshly prepared PANI/Pt FO-SPR sensors, with PANI in the Emeraldine form, were used for monitoring pH ranging from 7 to 1 (see Figure 6), and successively from 7 to 14 (see Figure 7). The FO-SPR spectral dips measured for selected acidic and alkaline pH values, are shown in Figure S1 from the Supplementary Materials file. Figure 6A presents as an example the SPR spectral dips obtained in a solution of pH 7 (black) and pH 1 (red), respectively, pointing out a clear wavelength red right-shift of the SPR dip with decreasing the pH value towards acidic solutions. This behavior can be naturally explained in terms of changing the optical properties of PANI sensitive film through its *RedOx* conversion from Emeraldine to Pernigraniline. As suggested elsewhere [32], along with the RI changes of the PANI film when exposed to the pH solutions, the molecular conformation of the PANI polymeric chains could be also slightly altered. This phenomenon is more pronounced in acidic media, and it may lead to noisier FO-SPR spectral dips, as observed in Figure 6A and Figure 7A. Figure 6B shows the SPR wavelength shift variation with the pH of the solution from 7 to 1. The sensitivity was calculated from the slope of this calibration curve and it was found to be 2.77 nm/pH ($R^{2}=0.95$).

Similarly, Figure 7A shows two SPR spectral dips obtained in the alkaline pH range, i.e., for pH 7 (black) and pH 14 (blue), respectively. In this case, the excess of $OH^{-}$ ions during PANI deprotonation leads to PANI Emeraldine-state reduction to Leucoemeraldine, causing a wavelength blue left-shift of the SPR dip with increasing the pH value towards basic solutions. The SPR wavelength shift variation with the pH of the solution from 7 to 14 is presented in Figure 7B and the sensitivity was calculated to be 3.18 nm/pH ($R^{2}=0.98$).

It can be, thus, concluded that the PANI/Pt-coated FO-SPR sensor provides a linear SPR response on the entire pH range, and that the observed pH response is mainly attributed to the sensitive RI changes of the PANI film, as the RI value of the pH solutions is maintained constant throughout the measurements [7,29]. In particular, in respect to the neutral pH value (7), it was observed that increasing the pH triggers a SPR wavelength shift towards lower wavelengths, whilst, oppositely, decreasing the pH determines a SPR wavelength shift towards higher wavelengths. The obtained sensitivity results in relation to the operational pH range, have been compared with other reported data achieved with various FO-SPR sensor’s configurations (see Table 1). The highest pH sensitivity, as claimed by S.K. Mishra et al. was found to be around 19.5 nm/pH, when employing a FO-SPR device with Ag/ITO/Al/hydrogel layer sequence [16]. However, the fabrication complexity of the sensor, as well as its slightly reduced pH operational range make this FO-SPR configuration less attractive. In contrast, other studies demonstrated lower sensitivity values ranging from 0.16 nm/pH to 2.48 nm/pH, only when operating in a narrow pH range [33,34]. It can be thus observed that the PANI/Pt-coated reflection-type FO-SPR sensor prepared and characterized in this work, provides a decent sensitivity value higher than 2.5 nm/pH on a broad range of pH values (1 to 14).

## 4. Conclusions

An innovative reflection-type PANI/Pt-coated FO-SPR pH sensor was fabricated and experimentally validated. The sensing area was based on a sensitive PANI film synthesized on the Pt-coated FO-SPR sensor through a simple cost-effective electroless procedure. The bulk sensitivity of the Pt-coated FO-SPR sensors was first evaluated in serial sucrose dilutions (of different RI units), owning to a value of 1734 nm/RIU, with a corresponding FOM around 9 ${\mathrm{RIU}}^{-1}$. Second, the as-prepared PANI/Pt-coated FO-SPR sensors showed encouraging results when employed for pH monitoring, with a sensitivity of 3.18 nm/pH for pH ranging from 7 to 14 and a sensitivity of 2.77 nm/pH for pH ranging from 7 to 1. These PANI/Pt-coated FO-SPR sensors may provide a broad interest for applications, since they offer many advantages like high sensitivity, quick response, easy handling, compactness, low-cost fabrication, and capability for remote sensing applications.

## Figures and Tables

**Table 1:** Sensitivity value and pH operational range reported while utilizing various FO-SPR pH sensor’s configurations.

FO-SPR pH Sensor Configuration	pH Operational Range	Sensitivity (nm/pH)
FO-SPR based on	3–11	19.5
Ag/ITO/Al/hydrogel layers [16]		
Nanoporous Poly(Ionic Liquid)	7–10	2.48
membrane on FO-SPR sensor [34]		
Fibre Bragg grating FO-SPR [33]	3–8	0.166
Reflection-type PANI/Pt-coated	1–7	2.77
FO-SPR sensor (present study)	7–14	3.18

## Data Availability

Not applicable.

## Supplementary Materials

The following are available online at https://www.mdpi.com/article/10.3390/s21124218/s1, Figure S1: Typical spectral dips obtained in (**A**) alkaline and (**B**) acidic pH solutions (ranging from pH 1 to pH 14), acquired with a reflection-type polyaniline/platinum (PANI/Pt) bilayer-coated Fiber Optic - Surface Plasmon Resonance (FO-SPR) sensor. The FO-SPR spectral dip corresponding to the neutral pH (7) is depicted in black.

## Abbreviations

The following abbreviations are used in this manuscript:

R&D	Research & Development
MDEO	R&D Center for Materials and Electronic & Optoelectronic Devices
IMCN	Institute of Condensed Matter and Nanosciences
UCLouvain	Université Catholique de Louvain
INFLPR	National Institute for Laser, Plasma and Radiation Physics
FO-SPR	Fiber Optic—Surface Plasmon Resonance
Pt, Au, Ag, Al	Platinum, Gold, Silver, Aluminum
ITO	Indium-doped tin oxide
PANI	Polyaniline
XPS	X-ray Photoelectron Spectroscopy
FE-SEM	Field Emission—Scanning Electron Microscope
DIW	Deionized water
RI(U)	Refractive index (units)
S, FOM	Sensitivity, Figure of Merit
FWHM	Full width at half-maximum
UEFISCDI	Executive Unit for Financing Higher Education, Research,
	Development and Innovation
BSMA	Bio- and Soft Matter Research Division
