# Supplementary material for: Sensitive pH Monitoring Using a Polyaniline-Functionalized Fiber Optic—Surface Plasmon Resonance Detector
Source: Sensors (Basel). 2021 Jun 19;21(12):4218. doi: 10.3390/s21124218 (PMC8235001; doi:10.3390/s21124218)
Supplement: Supplementary file 1 [file sensors-21-04218-s001.zip › sensors-1235287-supplementary.pdf]

sensors

Supplementary Information

Sensitive pH monitoring using a polyaniline-functionalized fiber optic–surface plasmon resonance detector

Iulia Antohe [1], Luiza-Izabela Jinga [1], Vlad-Andrei Antohe [2,3] and Gabriel Socol [1,*]

[1] National Institute for Laser, Plasma and Radiation Physics (INFLPR), Atomiștilor Street 409, 077125 Măgurele, Ilfov, Romania; iulia.antohe@inflpr.ro (I.A.); izabela.jinga@inflpr.ro (L.-I.J.)
[2] Faculty of Physics, R&D Center for Materials and Electronic & Optoelectronic Devices (MDEO), University of Bucharest, Atomiștilor Street 405, 077125 Măgurele, Ilfov, Romania; vlad.antohe@fizica.unibuc.ro
[3] Institute of Condensed Matter and Nanosciences (IMCN), Université Catholique de Louvain (UCLouvain), Place Croix du Sud 1, B-1348 Louvain-la-Neuve, Belgium
* Correspondence: gabriel.socol@inflpr.ro; Tel.: +40-(0)-21-457-4450 (ext. 2045)

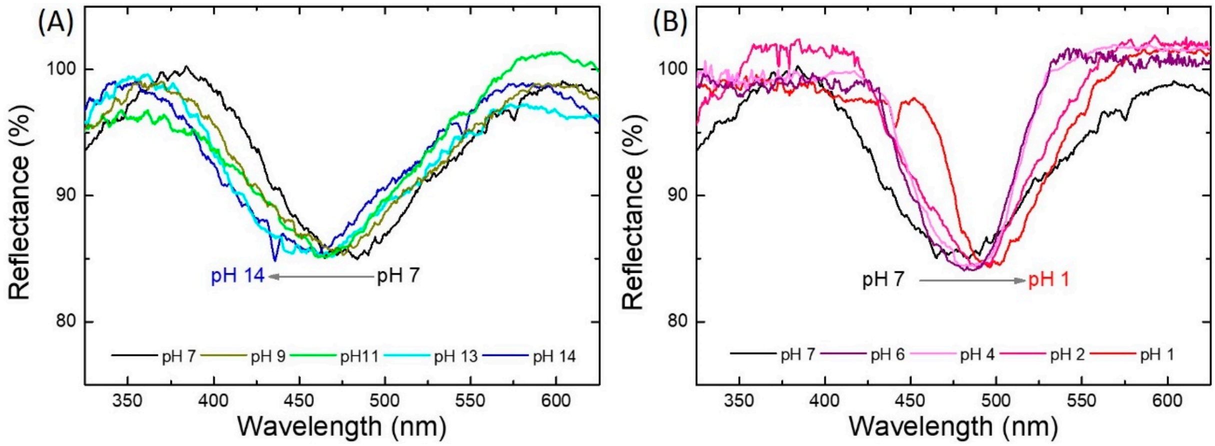

Figure S1. Typical spectral dips obtained in (**A**) alkaline and (**B**) acidic pH solutions (ranging from pH 1 to pH 14), acquired with a reflection-type polyaniline/platinum (PANI/Pt) bilayer-coated Fiber Optic – Surface Plasmon Resonance (FO-SPR) sensor. The FO-SPR spectral dip corresponding to the neutral pH (7) is depicted in black.
